# Diabetes experts' reasoning about diabetes prevention studies: a questionnaire survey

**DOI:** 10.1186/1756-0500-1-90

**Published:** 2008-10-14

**Authors:** Ingrid Mühlhauser

**Affiliations:** 1 Unit of Health Sciences, University of Hamburg, Hamburg, Germany

## Abstract

**Background:**

Presentation of results of diabetes prevention studies as relative risk reductions and the use of diagnostic categories instead of metabolic parameters leads to overestimation of effects on diabetes risk. This survey examines to what extent overestimation of diabetes prevention is related to overestimation of prevention of late complications.

**Methods:**

Participants of two postgraduate courses in clinical diabetology in Austria (n = 69) and Germany (n = 31) were presented a questionnaire with 8 items at the beginning of the meetings. All 100 questionnaires were returned with 92 filled in completely. Participants were asked 1) to rate the importance of differently framed results of prevention studies and, for comparison, of the United Kingdom Prospective Diabetes Study (UKPDS), 2) to estimate to what extent late complications could be prevented by the achieved reductions in diabetes risk or HbA1c values, respectively.

**Results:**

Prevention of diabetes by 60% was considered important by 84% of participants and 35% thought that complications could be prevented by ≥ 55%. However, if corresponding HbA1c values were presented (6.0% versus 6.1%) only 19% rated this effect important, and 12% thought that late complications could be prevented by ≥ 55%. The difference in HbA1c of 0.9% over 10 years in the UKPDS was considered important by 75% of participants and 16% thought that complications ('any diabetes related endpoint') were reduced by ≥ 55% (correct answer <15% by 20% participants).

**Conclusion:**

The novel key message of this study is that the misleading reporting of diabetes prevention studies results in overestimation of effects on late complications.

## Background

Diabetes health care professionals judge the benefit of preventive interventions substantially higher when changes in diabetes risk are communicated rather than related glycaemia parameters. [1]. In a recent study, we have surveyed participants of three European diabetes conferences. About 300 physicians and nurse educators were presented different formats of results of diabetes prevention studies. If results were communicated as a 57% reduction in diabetes risk about 90% of the diabetes experts interpreted the effect as important. In contrast, if the underlying changes of glucose or HbA1c values were presented, less than 40% rated the results as important [1]. Transformation of continuous metabolic data into diagnostic categories interferes with understanding of study effects.

Framing of data is a well recognised cause of misconceptions about efficacy of health interventions by physicians [2-5], patients [2,6], and health care decision makers [7]. This may be particularly relevant to preventive medicine [8,9]. Our survey has underscored that framing of data is also relevant for diabetes prevention [1].

However, it is not known why there are such impressive discrepancies in the ratings of the importance between changes in glycaemia parameters and risks of diabetes diagnosis. We hypothesised that diabetes health care providers may deduce from a 60% reduction in diabetes risk a comparably high reduction in diabetic late complications. Therefore, the aim of the present study was to further elucidate the framing of data in diabetes prevention and inferences by health care providers on the prevention of late complications.

## Methods

### Participants

The survey samples comprised participants of the 2007 EASD (European Association for the Study of Diabetes) Postgraduate Course in Evidence-based Clinical Diabetology in Austria (n = 69), and participants of the 2007 Annual Meeting of the Schleswig-Holstein Association of diabetologists in Northern Germany (n = 31). At both conferences diabetes prevention was a main topic. With few exceptions participants were certified diabetologists or other practising physicians with a special interest in diabetes care. The author was an invited speaker and not involved in the organisation of the meetings. She asked the audience for participation in the survey at the very beginning of the meetings. A questionnaire with 8 items was presented. In the Austrian meeting the questionnaires were analysed and descriptive results were presented and discussed at the end of the meeting. In the German meeting which lasted only half a day results of the Austrian meeting were presented and discussed. In order to achieve high participation both surveys were completely anonymous. Therefore, no further details on characteristics of participants were documented. Approval by an ethics committee was not considered necessary.

### Questionnaire

The questionnaire was in German language and based on the questionnaire which we had piloted and used in our first survey [1]. Three of the 8 items were related to the primary outcome ('any diabetes related endpoint') of the UKPDS [10]. The other five items referred to primary diabetes prevention trials [11-14]. Details on the publications which formed the basis of the questionnaires and the construction of the first questionnaire have been described previously [1]. To minimise bias due to the sorting of items three versions of the questionnaire with different orders of items were used (A, B, C). The translated questionnaire (version A) with an explanation on how alternative presentations were derived is provided as additional file 1. Two items (1 and 6) were similar to the previous survey [1] to allow an estimate on the reproducibility of results. Identical wording was used in all items rating effects on diabetes risks or the primary outcome in the UKPDS: "How important do you consider the benefit of this intervention?" Participants were asked to mark their ratings as either "very important", "important", "not very important" or "not important at all". Wording on the effects related to prevention of late complications was also identical for all related items: "What is your estimate? On the long-term late complications could be reduced by ... " and participants were asked to score one answer among the following: "more than" 75%, "55 to 75%", "35 to 55%", "15 to 35%", "less than 15%".

### Assumptions

Based upon a vast literature on framing of data [2-7] and the results of our previous survey [1] the following assumptions were made: 1) Items similar to those of the first survey (items 1 and 6) achieve comparable ratings as in the previous study [1]. 2) Effect sizes are rated highest when presented as relative risk reduction (item 1), followed by decreasing importance when results are presented as HbA1c values (item 3) or natural frequencies (item 8) for the UKPDS primary endpoint, gain in life expectancy as estimated for primary prevention studies (item 5), and of lowest importance when glycosylated haemoglobin values (HbA1c) are presented for life style interventions in the primary diabetes prevention studies (item 6). 3) The extent to which late complications could be prevented would be rated highest for a 60% reduction of diabetes risk (item 2), lowest for a change in HbA1c values from 6.0% to 6.1% (item 7), and in between for the reduction of HbA1c by almost 1% over 10 years in the UKPDS (item 4).

### Data collection and analysis

Data were analysed using SPSS, version 15.0 for windows. Comparable to our previous survey [1] only descriptive data are presented. No statistical comparisons between subgroups were made.

## Results

**Figure 1:**
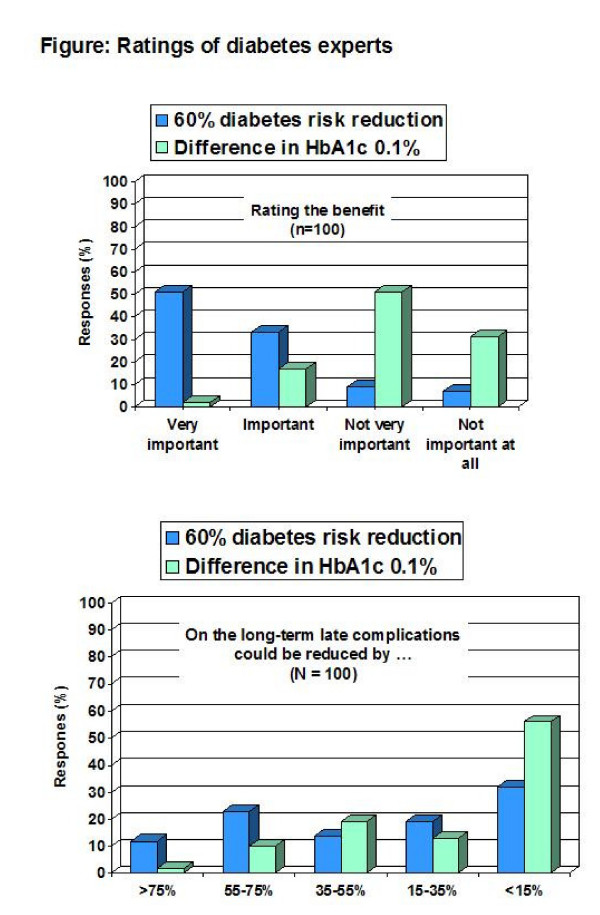
Ratings of 100 diabetes experts on the benefit of diabetes prevention interventions on diabetes risk (upper panel) and estimates on corresponding reductions of diabetic late complications (lower panel) in relation to the presentation of the results as either a 60% diabetes risk reduction or the corresponding difference in HbA1c values of 0.1% (6.0% versus 6.1%).

All 100 distributed questionnaires were returned, and except 8 all were filled in completely. Predefined assumptions were supported by the survey results (Table 1). Results were comparable across the two survey subpopulations (Table 1 ) and the three versions of the questionnaire (data not shown). 84% of participants considered a 60% reduction in diabetes risk as important (51% as "very important") but only 19% (2%) when a change in HbA1c of 0.1% was presented (Figure 1). When the result was presented as a 60% reduction in diabetes risk 35% of participants expected a reduction of long-term complications by ≥ 55% but only 12% did so when a decrease in HbA1c of 0.1% was used (Figure 1). The presentation of expected life years gained yielded results in between (Table 1).

The difference in HbA1c of 0.9% over 10 years in the UKPDS was rated important by 75% (by 35% as "very important"). 16% thought that complications were reduced by ≥ 55% (43% thought by ≥ 35%, correct <15%). When the corresponding results of the number of patients with a primary outcome event were presented (41 versus 46 of 100) only 9% considered this effect as "very important" (Table 1).

**Table 1:** Ratings of diabetes experts of study effects and associated risk reductions of late complications in relation to format of data presentation

	**Rating the benefit of the intervention as**				**Estimates of corresponding reductions of late complications**				
	very important	important	not very important	not important at all	>75%	55–75%	35–55%	15–35%	<15%
**Diabetes prevention**									

60% diabetes risk reduction (item 1 and 2)	35/15 (51%)	19/14 (33%)	8/1 (9%)	6/1 (7%)	7/5 (12%)	14/8 (23%)	9/5 (14%)	11/7 (19%)	25/6 (32%)
HbA1c 6.0% versus 6.1% (item 6 and 7)	1/1 (2%)	10/7 (17%)	35/15 (51%)	22/8 (30%)	1/1 (2%)	8/2 (10%)	14/5 (19%)	8/5 (13%)	37/18 (56%)
0.288 life years gained (item 5)	3/3 (6%)	17/9 (27%)	34/15 (50%)	13/4 (17%)	n.a.	n.a.	n.a.	n.a.	n.a.

**UKPDS**									

HbA1c 7.0% versus 7.9% (item 3 and 4)	22/12 (35%)	24/15 (40%)	21/4 (25%)	0/0	2/2 (4%)	7/4 (12%)	20/6 (27%)	22/13 (37%)	13/6 (20%)
41 versus 46 out of 100 patients (item 8)	5/4 (9%)	36/17 (54%)	25/10 (36%)	1/0 (1%)	n.a.	n.a.	n.a.	n.a.	n.a.

Values are numbers of the n = 69 Austrian/n = 31 German samples (percentages of all respondents). UKPDS = United Kingdom Prospective Diabetes Study [10]. Numbers may not add up to 100 due to up to 5 missing values. n.a. = not assessed

## Discussion

The present study supports and extends the findings of our previous survey [1]. Diabetes experts overestimate the benefit of preventive interventions when outcomes are presented as changes in diabetes risk rather than real metabolic changes. In addition, the findings further elucidate the reasoning of physicians confronted with different outcome descriptions. From a high reduction in diabetes risk they wrongly infer a high reduction of late complications. On the other hand, when the corresponding results of HbA1c values of the same large randomized trials are presented they expect a much lower reduction of late complications. Therefore, the original hypothesis of this survey is supported. Overall, reductions of late complications were substantially overestimated irrespective of outcome presentation although overestimation was highest when results were presented as reductions in diabetes risk and lowest when HbA1c vales were presented supporting the predefined assumptions of this study.

The diabetes prevention studies included individuals with elevated fasting and post-load glucose concentrations who were already at the brink of diabetes [11-13,15,16]. Therefore, minimal differences in fasting plasma glucose of 0.3 mmol/L or HbA1c values of 0.1% may relate to pronounced differences in proportions of persons with a diagnosis of diabetes and diabetes risk reductions. In the Diabetes Prevention Program life style changes reduced the risk of diabetes by about 50% after 3 years. However, the corresponding glycaemia changes were modest. After 3 years mean glycosylated haemoglobin was about 6.0% in the lifestyle intervention group and 6.1% in the control group as estimated from figure 1 of the publication [13]. Small metabolic differences are magnified by transformation of continuous data into categorical data [1,17].

Cutoffs for plasma glucose to diagnose diabetes have been repeatedly challenged as there is no abrupt glycaemic threshold above which diabetes complications increase [18]. Therefore, transition from impaired glucose tolerance to diabetes may be considered as a change in diagnostic labelling rather than change from a health to a disease state. Cost-effectiveness analyses have estimated that the Diabetes Prevention Program life style intervention would reduce a high-risk person's 30-year chances of a serious complication from about 38% to 30% which relates to an absolute risk reduction of 8% and a relative risk reduction of 21% [14]. In some of the publications of the primary prevention studies crucial metabolic data are not communicated or difficult to extract [1]. The Finish Diabetes Prevention Study included HbA1c as a secondary outcome measure [19] but did not report results in the main publications [11,20]. Neither blood glucose nor HbA1c values have been reported in the core publication of the STOP-NIDDM Acarbose prevention study [15]. Despite our previous survey [1] meta-analyses of diabetes prevention studies continue to be published without reporting or commenting on metabolic parameters [21]. Incomplete reporting of outcomes within published articles of randomised trials is common and is associated with statistical non-significance [22]. Biased reporting and framing of data by researchers and authors may enhance misconceptions about treatment effects among users of study results [1-7].

The present study has limitations. The study population is a convenience sample and includes only German speaking diabetes experts whereas the previous survey included both nurses and physicians of international diabetes meetings. In contrast to our first survey detailed characteristics of participants were not assessed in the present study. However, the previous survey also included a well characterised subgroup of 101 German diabetes experts who were participants of the 2005 EASD (European Association for the Study of Diabetes) Postgraduate Course in Evidence-based Clinical Diabetology in Jena, Germany, an equivalent of the Austrian meeting evaluated in the present survey. Among the Jena sample of diabetes experts about 40% worked at primary-care centres, 19% at non-university hospitals, 27% at university or research institutions, and the rest at other institutions. On average, they had worked for about 9 years in clinical diabetology and 57% reported to have participated in a course of evidence-based medicine [1]. Results between both surveys were comparable on related items. In our previous study a reduction in diabetes risk of about 60% was rated important by 92% and a difference in HbA1c between 6.0% and 6.1% by 18% of participants [1] compared to 84% and 19%, respectively, in the present survey. In both surveys we have used data from the UKPDS. The UKPDS is a landmark study familiar to all diabetologists. Nevertheless, in our previous survey only 6 out of 299 diabetologists and diabetes educators could approximately state the frequencies for the primary outcome (41 versus 46 out of 100). Of those who responded most participants substantially overestimated the intervention effect. Also, in the present survey the effect of the intervention was overestimated. Only 20% gave the correct answer (less than 15%). Despite these similarities between the two surveys results of the present study may not be representative for other countries and other groups of physicians or health care providers.

## Conclusion

This survey provides further insights into the reasoning of physicians. Diabetes experts overestimate the benefit of diabetes prevention studies irrespective of outcome presentation. The novel key message of this study is that from a high reduction of diabetes risk they wrongly infer a high reduction of late complications. The findings again urge presentation of study results in a format that can be understood by health care professionals. Understanding of prevention studies is fundamental for appropriate allocation of resources.

## Abbreviations

UKPDS: United Kingdom Prospective Diabetes Study; HbA1c: glycosylated haemoglobin.

## Competing interests

The author declares that she has no competing interests.

## Supplementary Material

Additional File 1 The file provides the translation of the original German questionnaire (version A) and explanations on how alternative presentations were derived. Click here for file
