# Supplementary material for: Diabetes experts' reasoning about diabetes prevention studies: a questionnaire survey
Source: BMC Res Notes. 2008 Oct 14;1:90. doi: 10.1186/1756-0500-1-90 (PMC2588450; doi:10.1186/1756-0500-1-90)
Supplement: Additional File 1 — The file provides the translation of the original German questionnaire (version A) and explanations on how alternative presentations were derived. [file 1756-0500-1-90-S1.pdf]

Translation of the original German questionnaire (version A)

Dear participants,

several randomised controlled trials on diabetes prevention have been published. They have investigated life style and drug interventions. The results have been presented at congresses and reported in the media. What is your impression about the significance of these interventions?

Please mark your ratings (one answer only):

1) In one of the diabetes prevention studies manifestation of diabetes could be reduced by almost 60%. How important do you consider the benefit of this intervention?
The benefit is

- very important
- important
- not very important
- not important at all

2) What would be the long-term benefit of this intervention with a diabetes reduction of 60% on the **prevention of late complications**? What is your estimate?
On the long-term late complications could be reduced by

- more than 75%
- between 55 and 75%
- between 35 and 55%
- between 15 and 35%
- less than 15%

3) In the UKPDS there was a statistically significant reduction of late complications in the patients with type 2 diabetes. With intensified diabetes therapy HbA1c values were 7.0% compared to 7.9% with conventional therapy.
How important do you consider the benefit of this intervention?
The benefit is

- very important
- important
- not very important
- not important at all

4) What is your estimate on the benefit of this improvement of HbA1c values by almost 1% in the UKPDS on late complications (the primary endpoint “any diabetes related endpoint”)?
In the UKPDS late complications could be reduced by

- more than 75%
- between 55 and 75%
- between 35 and 55%
- between 15 and 35%
- less than 15%

5) A continuous long-term diabetes prevention programme would have the following effects on the remaining life expectancy: with standard treatment it would be a mean of 24.032 years, with the diabetes prevention programme there would be a gain of additional 0.288 years (a statistically significant difference).
How important do you consider the benefit of this intervention?
The benefit is

- o very important
- o important
- o not very important
- o not important at all

6) In one of the diabetes prevention studies after 3 years the HbA1c value was 6.0% in the intervention group, and 6.1% in the control group.
How important do you consider the benefit of this intervention?
The benefit is

- o very important
- o important
- o not very important
- o not important at all

7) What would be the long-term benefit of this intervention with a reduction of HbA1c by 0.1% on the **prevention of late complications**? What is your estimate?
On the long-term late complications could be reduced by

- o more than 75%
- o between 55 and 75%
- o between 35 and 55%
- o between 15 and 35%
- o less than 15%

8) Intensified blood glucose control in patients with type 2 diabetes over 10 years would show the following results with respect to late complications:
With intensified therapy 41 of 100 patients would experience late complications, with standard therapy there would be 46 of 100 patients with late complications.
How important do you consider the benefit of this intervention?
The benefit is

- o very important
- o important
- o not very important
- o not important at all

Many thanks!

Explanations on how alternative presentations are derived

Items 1 and 6 are based on study results for the life style intervention arms of primary diabetes prevention studies [1, 2]. In the Finish Diabetes Prevention Study [1] life style changes reduced the risk of diabetes by almost 60% after 2 years (item 1), and in the Diabetes Prevention Program by about 50% after 3 years. However, the corresponding glycaemic changes were modest. In the Finish Diabetes Prevention Study [1] the mean change from baseline in the fasting plasma glucose concentration was -2 mg/dl (-0.1 mmol/L) in the intervention group and +3 mg/dl (+0.2 mmol/L) in the control group after two years. Changes for HbA1c values were not reported in the Finish Diabetes Prevention Study [1,3]. For the Diabetes Prevention Program we have estimated glycosylated haemoglobin values from figure 3 of the publication [2]. After 3 years mean glycosylated haemoglobin was about 6.0% in the lifestyle intervention group and 6.1% in the control group (item 6).
The diabetes prevention studies included individuals with elevated fasting and post-load glucose concentrations who were already at the brink of diabetes. Therefore, minimal differences in fasting plasma glucose of 0.3 mmol/L or HbA1c values of 0.1% may relate to pronounced differences in proportions of persons with a diagnosis of diabetes and diabetes risk reductions of more than 50%. Small metabolic differences are magnified by transformation of continuous data into categorical data.
Items 2 and 7 refer to the related changes in late complications. Since long-term randomized controlled trials have not been performed data on late complications are not available. However, cost-effectiveness analyses have estimated that the Diabetes Prevention Program life style intervention would reduce a high-risk person's 30-year chances of a serious complication from about 38% to 30% which relates to an absolute risk reduction of 8% and a relative risk reduction of 21% [4]. After 30 years the expected life-years gained by a life style program were estimated to be 0.288 years with a mean life expectancy of 24.032 years without lifestyle program [4]. Item 5 addresses this outcome.
Items 3, 4, and 8 refer to the UKPDS main results on the primary endpoint [5]. The risk reduction for the primary end point ("any diabetes related endpoint" – a composite endpoint mainly of serious complications) in the UKPDS was 12% (95% CI 1-21%, p=0.029) with a number needed to treat for 10 years of 20 (95% CI 10 to 500). In the conventional treatment group with a median HbA1c over 10 years of 7.9%, 46 out of 100 patients had a primary endpoint, and in the intensified treatment group with a median HbA1c of 7.0%, 41 out of 100 patients suffered any diabetes related endpoint.

References

1. Tuomilehto J, Lindström J, Eriksson J-G et al. (2001) Prevention of type 2 diabetes mellitus by changes in lifestyle among subjects with impaired glucose tolerance. NEJM 344:1343-50
2. Diabetes Prevention Program Research Group (2002) Reduction in the incidence of type 2 diabetes with lifestyle intervention or metformin. NEJM 346:393-403
3. Lindström J, Parikka PI, Peltonen M et al. Sustained reduction in the incidence of type 2 diabetes by lifestyle intervention: follow-up of the Finnish Diabetes Prevention Study. Lancet 2006;368:1673-9
4. Eddy DM, Schlessinger L, Kahn R. Clinical outcomes and cost-effectiveness of strategies for managing people at high risk for diabetes. Ann Intern Med 2005;143:251-64
5. UK Prospective Diabetes Study (UKPDS) Group (1998) Intensive blood-glucose control with sulphonylureas or insulin compared with conventional treatment and risk of complications in patients with type 2 diabetes (UKPDS 33). Lancet 352:837-53
